# Testosterone Increases Circulating Dehydroepiandrosterone Sulfate Levels in the Male Rhesus Macaque

**DOI:** 10.3389/fendo.2014.00101

**Published:** 2014-06-25

**Authors:** Krystina G. Sorwell, Steven G. Kohama, Henryk F. Urbanski

**Affiliations:** ^1^Division of Neuroscience, Oregon National Primate Research Center, Beaverton, OR, USA; ^2^Department of Behavioral Neuroscience, Oregon Health & Sciences University, Portland, OR, USA; ^3^Department of Physiology and Pharmacology, Oregon Health & Sciences University, Portland, OR, USA; ^4^Division of Reproductive and Developmental Sciences, Oregon National Primate Research Center, Beaverton, OR, USA

**Keywords:** adrenal gland, aging, androgen, dehydroepiandrosterone, non-human primate, testosterone

## Abstract

The adrenal steroid dehydroepiandrosterone (DHEA) and its sulfate (DHEAS) are two of the most abundant hormones in the human circulation. Furthermore, they are released in a circadian pattern and show a marked age-associated decline. Adult levels of DHEA and DHEAS are significantly higher in males than in females, but the reason for this sexual dimorphism is unclear. In the present study, we administered supplementary androgens [DHEA, testosterone and 5α-dihydrotestosterone (DHT)] to aged male rhesus macaques (*Macaca mulatta*). While this paradigm increased circulating DHEAS immediately after DHEA administration, an increase was also observed following either testosterone or DHT administration, resulting in hormonal profiles resembling levels observed in young males in terms of both amplitude and circadian pattern. This stimulatory effect was limited to DHEAS, as an increase in circulating cortisol was not observed. Taken together, these data demonstrate an influence of the hypothalamo-pituitary–testicular axis on adrenal function in males, possibly by sensitizing the zona reticularis to the stimulating action of adrenocorticopic hormone. This represents a plausible mechanism to explain sex differences in circulating DHEA and DHEAS levels, and may have important implications in the development of hormone therapies designed for elderly men and women.

## Introduction

Levels of the adrenal steroid dehydroepiandrosterone (DHEA) and its sulfate (DHEAS; collectively referred to as DHEA/S) differ between males and females, yet the underlying cause for this sexual dimorphism is unknown (1). It is clear, however, that the gross, cellular, and molecular adrenal structure of males and females is essentially similar, suggesting that some factor outside the adrenal gland is responsible for the greater secretion of DHEA/S in males (2). Given that the sex differences in DHEA/S are most pronounced during early adulthood, it is plausible that the different levels of sex-steroid hormones between adult males and females may play a contributing role. In fact, previous work in perimenopausal women and macaque models of menopause has shown that decreasing levels of estrogens correspond to increases in DHEAS (3–5), and estrogen replacement paradigms decrease DHEA/S (5, 6) at a rate greater than seen in normal aging without estrogen supplementation. As DHEA/S is a precursor to estradiol (7), this may reflect a compensatory interaction between the hypothalamic–pituitary–gonadal (HPG) and hypothalamic–pituitary–adrenal axes. On the other hand, males have higher circulating levels of DHEAS than do females, despite having substantially higher levels of testosterone (T). This suggests that an inverse relationship between adrenal and gonadal steroids exists in females, whereas a positive causal relationship may exist in males.

The hormone systems discussed so far not only have the potential for interacting with each other, but they also show significant age effects. In both sexes, DHEA/S declines gradually and consistently starting in early adulthood, with levels reaching roughly 40% of their peak by the middle age and continuing to decline thereafter (8, 9). In females, menopause is associated with a sudden decline in estrogen (10, 11), while males show a less precipitous decline in testosterone during aging. The situation is further complicated by therapeutic hormonal supplementation in the elderly, which can alter circulating sex-steroid hormone levels, though not necessarily in the most appropriate physiological manner. Furthermore, in the USA DHEA is sold as a food supplement, requiring no prescription, and so is widely used by the elderly for self-medication. Thus, to fully understand the physiological impact of hormone changes with age the interactions between the HPG and HPA axes require further investigation under carefully controlled experimental conditions, something that can be achieved more readily using non-human primates such as the rhesus macaque.

Physiological replacement of steroids may protect against many negative aspects of aging, but it is first important to understand the differences between males and females and how these systems interact to determine the safest and most effect form of hormone therapy (HT). We used the rhesus macaque, a large diurnal primate with endocrine physiology very similar to that of humans, to investigate the interactions between DHEA/S and testosterone. Because we recently observed novel interactions in a combined androgen supplementation paradigm (12, 13), we have expanded on this previous research to formulate a working hypothesis for adrenal and gonadal interactions. Our results indicate a strong influence of testosterone on circulating levels of DHEAS, possibly explaining the drastic differences between male and female levels of the hormone.

## Materials and Methods

### Animals

The study used adult rhesus macaques (*Macaca mulatta*), and was approved by the OHSU Institutional Animal Care and Use Committee. The animals were cared for by the Division of Comparative Medicine at the Oregon National Primate Research Center (ONPRC) in accordance with the *National Research Council’s Guide for the Care and Use of Laboratory Animals*. They were caged singly indoors under controlled environmental conditions: 24**°**C temperature; 12-h light, 12-h dark photoperiods (lights on at 0700 h). Monkey chow was provided at 0800 and 1500 h and was supplemented with fresh fruit and vegetables; fresh drinking water was available *ad libitum*.

In Experiment 1, ten adult (11–12 years) male and eight adult (11–12 years) female rhesus macaques were used to assess sex differences in circulating DHEAS levels. In Experiment 2, four young adult (7–12 years) and four old (21–26 years) males were used to examine age-related changes in circulating DHEAS levels; five additional old males were used to evaluate the impact of androgen supplementation on DHEAS.

### Remote blood sampling

Cortisol and DHEA/S both demonstrate a circadian pattern of release (14–16). Therefore, to gain meaningful insights regarding sex-related or age-related hormone differences it was necessary to collect blood samples from each animal serially across an entire 24-h period. To achieve this with minimal disruption of the animals, each monkey was surgically implanted with a subclavian vein catheter, leading to a remote blood sampling system in an adjacent room, as previously described (17). Blood samples were collected into EDTA-coated borosilicate glass tubes every hour for a complete 24-h cycle. The samples were centrifuged at 4**°**C, and the plasma was stored at −20**°**C until assay for cortisol and DHEAS. Cortisol was assayed using electrochemiluminescence using the Elecsys 2010 Platform (Roche Diagnostics, Indianapolis, IN, USA). DHEAS was assayed using radioimmunoassay with a highly specific antibody for DHEAS-17-(O-carboxymethyl)oxime-BSA (Endocrine Services, Tarzana, CA, USA) and [^3^H]DHEAS (SA, 22 Ci/mmol). Intra- and interassay coefficients of variation were less than 10% for each assay and the assay detection limits were 3 ng/ml. The 24-h serial blood sampling procedure was performed once on each animal, except for the old androgen-supplemented animals, which were re-sampled after each of the 5-day androgen supplementation tests, performed approximately 1 month apart (Table 1). Data were analyzed using a repeated-measures ANOVA with time as a within-subjects factor and group (young, old baseline, old supplemented) as a between-subjects factor. When the assumption of sphericity was not met, a Greenhouse-Geisser correction was implemented.

**Table 1:** **Steroid supplementation paradigms**.

	Time of hormone administration		
Experiment	0700 h	1000 h	1900 h
Baseline	–	–	–
1	DHEA (0.04 mg/kg)	DHEA (0.04 mg/kg)	T (12 mg/kg)
2	DHEA (0.10 mg/kg)	DHEA (0.05 mg/kg)	–
3	DHEA (0.10 mg/kg)	DHEA (0.05 mg/kg)	DHT (5–10 mg/kg)
4	T (12 mg/kg)	–	–

*The times and doses of the four androgen supplementation paradigms are shown. Due to limitations on blood sampling, not all combinations of hormones were able to be tested at all doses. DHEA and T were dissolved in sesame oil, and were mixed into chocolates for oral supplementation. Animals were monitored to ensure the entire dose was eaten in a timely manner*.

### Steroid supplementation

Animals were treated with hormones for 5 days prior to each blood sampling session to allow steroid levels to equilibrate. DHEA (10 mg/ml; Sigma-Aldrich, St. Louis, MO, USA), testosterone (T, 120 mg/ml; Sigma-Aldrich), and dihydrotestosterone (DHT, 5–10 mg/ml; Sigma-Aldrich) were dissolved in commercial food-grade sesame oil and mixed with melted chocolate, a preferred treat. The steroids were dissolved in sesame oil to reduce their uptake and rapid metabolism by the liver and instead by increasing their absorption through the lymphatic system (18, 19). Chocolates were kept refrigerated at 4**°**C until the time of administration. Animals were watched at the time of administration to ensure the entire treat was eaten. To isolate potential mechanisms of adrenal androgen interaction, four supplementation paradigms were performed (Table 1): (1) DHEA administered at 0700 and 1000 h, T administered at 1900 h; (2) DHEA administered at 0700 and 1000 h; (3) DHEA administered at 0700 and 1000 h, DHT administered at 1900 h; and (4) T administered at 0700 h. These doses were selected based on preliminary experiments that were aimed at replicating the hormone levels observed in young adults; consequently, the doses varied between experiments. To protect animals against excessive blood sampling, it was not feasible to repeat all hormone combinations at all doses in all of the animals. Times were chosen to replicate the endogenous circadian peaks of T and DHEA, while in the last experiment T was administered in the morning to examine a possible role of time of day on the steroid response to T. Animals were monitored at the time of steroid supplementation to ensure each ate the entire treat in a timely manner; if the treat was refused, an equivalent dose was administered via a steroid-soaked cookie or prune. The doses used for each experiment are provided in the respective figures. As shown previously (13), the dose paradigm used in Experiment 1 is sufficient to restore circulating T and DHT to levels seen in young adult male rhesus macaques.

## Results

### Young adult male rhesus macaques show significantly higher levels of DHEAS than females

**Figure 1:**
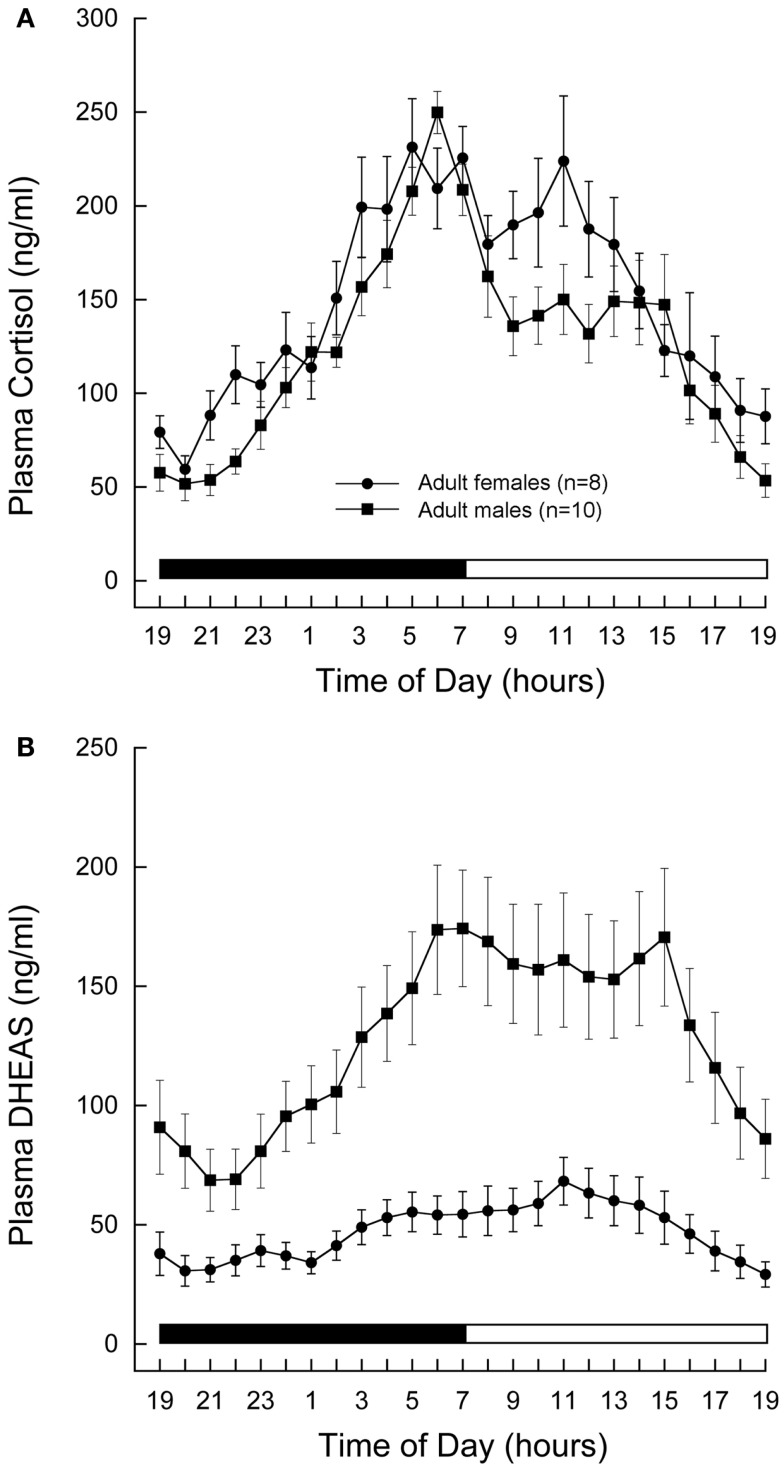
**Twenty-four-hour plasma profiles of adrenal steroids in male and female rhesus macaques**. Serial blood samples were remotely collected from adult (11–12 years) male (*n* = 10) and female (*n* = 8) animals and assayed for cortisol **(A)** and DHEAS **(B)**. Each data point represents the mean and vertical lines indicate the SEM. Time of day is indicated along the abscissa, and periods of darkness and light are represented by black and white bars, respectively. In both sexes, cortisol and DHEAS levels rose gradually during the night and reached peak levels around the time of lights on in the morning. Mean cortisol levels were similar in the two sexes, whereas mean DHEAS levels were significantly higher in males than in females (*P* < 0.01, Student’s *t*-test.)

Cortisol and DHEAS showed well-defined 24-h plasma profiles, both in the males and females (Figures 1A,B, respectively). The plasma levels rose gradually during the night and reached a peak in the morning at about the time when the lights came on. Although mean cortisol levels were similar in the two sexes, mean DHEAS levels were significantly higher in males than in females (*P* < 0.01, Student’s *t*-test).

### Aging is associated with decreased circulating DHEAS levels

**Figure 2:**
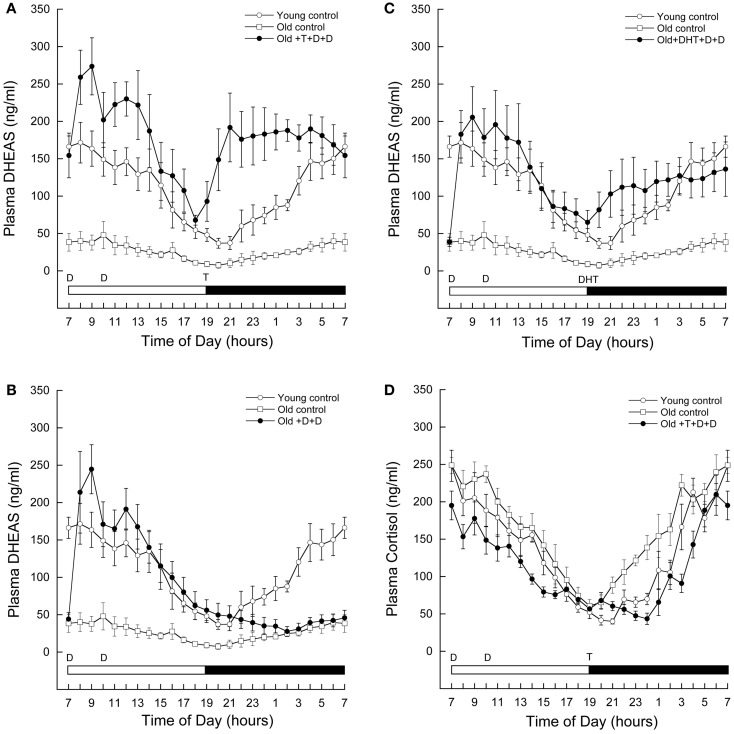
**Age-related changes in circulating adrenal steroid levels in male rhesus macaques, and the impact of androgen supplementation**. Serial blood samples were remotely collected from young (7–12 years, *n* = 4) and old (21–26 years, *n* = 4) males, as well as old males exposed to 5 days of various androgen supplementation paradigms (21–26 years, *n* = 5). The samples were subsequently assayed for DHEAS **(A–C)** and/or cortisol **(D)**. Each data point represents the mean and vertical lines indicate the SEM. Time of day is indicated along the abscissa, and periods of light and darkness are represented by white and black bars, respectively. In each panel, the times of oral androgen administration are depicted as follows: D = DHEA (0.04–0.10 mg/kg body weight), T = testosterone (12 mg/kg body weight), DHT = 5α-DHT (5–10 mg/kg body weight). For reference, the same DHEAS profiles from the young and old controls are depicted in **(B–C)**. The data demonstrate that androgen supplementation at an appropriate time of day can restore 24-h circulating DHEAS levels in old males rhesus macaques. Importantly, they also demonstrate an unexpected stimulatory action of gonadal steroids on DHEAS.

As shown in Figure 2A, circulating levels of DHEAS at baseline were significantly lower in aged (open squares) than in young males (open circles). A repeated-measures ANOVA with time as within-subjects factor and group (young and old baseline) as between-subjects factor indicated a significant effect of time (*F* = 6.427, *P* < 0.001), significant effect of group (*F* = 21.828, *P* = 0.003), and a significant group-by-time interaction (*F* = 7.146, *P* = 0.001). Circulating DHEAS was higher in young males at all time points.

### Testosterone administration in aged male rhesus macaques significantly increases circulating DHEAS to levels observed in young male rhesus macaques

Supplementation with DHEA resulted in a significant increase in circulating DHEAS in old males shortly after administration (Figure 2A, closed circles). Interestingly, although DHEAS levels declined throughout the day, they again began to increase shortly after administration of T at 1900 h. This increase was sustained throughout the night, resulting in higher DHEAS levels than baseline in the morning before DHEAS had been administered.

The supplementation paradigm was modified to further explore this phenomenon. If DHEAS rose throughout the night in the absence of exogenous T, we could conclude that this is a possible priming effect, by which exogenous DHEA stimulates the adrenal glands to produce more of their own DHEAS for the following day. However, as shown in Figure 2B, circulating DHEAS remained low throughout the night when no T was administered. A repeated-measures ANOVA with Greenhouse-Geisser correction for differences in sphericity was performed, comparing DHEAS between 1900 and 0700 h in animals receiving both T and DHEA as described versus animals receiving DHEA in the morning but no T at 1900 h. This test revealed a significant effect of time (*F* = 4.978, *P* = 0.026), treatment (*F* = 20.786, *P* = 0.003), and treatment-by-time interaction (*F* = 10.640, *P* = 0.002).

### DHT administration in aged male rhesus macaques significantly increases circulating DHEAS to levels seen in young male rhesus macaques

The results from the previous experiments suggest that T itself can increase circulating DHEAS. Testosterone is only two steps beyond DHEA in the steroidogenic pathway, and our dose of T needed to be very high in order to increase circulating levels to those of young animals. Therefore, we hypothesized that some of the exogenous T may have been back-converted to DHEAS, resulting in the observed gradual increase in circulating DHEAS levels. To test this possibility we replaced the exogenous T in our androgen supplementation paradigm with its more active metabolite, DHT. We reasoned that if our administered T was being converted back to DHEAS, then substitution of T with DHT, a hormone further along the steroidogenic pathway, should prevent the increase in DHEAS levels from occurring. However, the results clearly show that even with this alternate androgen supplementation paradigm circulating DHEAS increased above baseline levels starting at 1900 h; the resulting hormone pattern was nearly identical to that of young control animals (Figure 2C). A repeated-measures ANOVA between the baseline and DHT-supplemented animals revealed a significant effect of group (*F* = 12.944, *P* = 0.009), with DHT treatment resulting in an increase in circulating DHT starting at 1900 h. These data suggest that not only is a back-conversion of T to DHEA/S unlikely, but that the increase in circulating DHEAS is an androgen-receptor-mediated mechanism.

### Testosterone and DHEA supplementation do not cause an increase circulating cortisol

Both DHEA and cortisol are secreted by the adrenal gland in response to adrenocorticotropic hormone (ACTH) from the hypothalamus. To further examine where testosterone is having an effect to increase DHEAS, we also assayed a series of 24 h blood samples from our DHEA and T combined supplementation paradigm for cortisol. If androgen activation at the level of the brain increases ACTH secretion, cortisol would also increase at the time of T supplementation. However, as shown in Figure 2D, T supplementation did not result in increased circulating cortisol levels. A repeated-measures ANOVA with time as a within-subjects factor and group (old control, open circles; old androgen-treated, closed circles; and young, open squares) as a between subjects factor revealed a significant effect of time (*F* = 56.758, *P* < 0.001), group (*F* = 5.438, *P* = 0.028), and group-by-time interaction (*F* = 2.093, *P* < 0.001). The significant effect of group was driven by differences between the old control and old androgen-treated animals, with androgen treatment associated with significant reductions in circulating cortisol at 1000, 1500, 2100, 2200, 2300, 2400, and 0300 h. There was no significant difference between circulating cortisol in the young animals as compared to either the old control or old androgen-treated animals.

## Discussion

Research on adrenal sex differences in humans and non-human primates is limited, but some interesting observations have been made. The finding that DHEA/S differs dramatically between males and females is highly consistent and is maintained throughout the lifespan in both humans and rhesus macaques (7, 20–22), but to date no theories as to the mechanism of this difference have been adequately investigated. The differences in aging profiles of adrenal and gonadal hormones further complicates any potential interactions, as estrogen in females drops precipitously at the time of menopause, DHEA/S in both sexes declines slowly and consistently starting in the third decade (8), and testosterone in males decreases very slowly, gradually, and to a much lesser extent than other hormones (23–25).

While much work is yet to be done to study differences between the male and female adrenal gland, early work suggests that the difference in adrenal output is not due to intrinsic physiological differences but due to differential hormonal input to the adrenal glands of males and females. Although sex differences in both circulating DHEA/S and adrenal morphology and physiology are seen in the marmoset, with respect to both size of the zona reticularis (ZR, the layer of the adrenal gland that synthesizes DHEA/S) and expression of steroidogenic genes, similar differences are not observed in humans. Specifically, in the marmoset, females secrete significantly more DHEA/S than males due to both gross anatomical differences in the adrenal gland with increased adrenal zonation, as well as differences in enzymatic machinery with an increased expression of cytochrome b5 (26). However, in humans the male and female ZR are similar in both size and cytochrome b5 expression (27). Also, cultured male and female adrenal glands respond with identical levels of DHEA secretion when stimulated by ACTH (2). Further, females receiving long-term treatment with T show an increased response of DHEA to ACTH stimulation as compared to female controls (28, 29), suggesting that the adrenal machinery is the same in males and females, but the hormonal input from the HPG axis can modulate adrenal output. Given the established research on male and female adrenal physiology and the lack of evidence for sex differences in either gross morphology or cellular physiology, it is our hypothesis that the higher level of DHEAS seen in male humans and non-human primates is due to T increasing the sensitivity of the ZR to ACTH. Consistent with this hypothesis, our paradigm of testosterone supplementation significantly increased circulating DHEAS of aged male macaques in a manner mimicking the circadian profile of DHEAS in young male macaques.

Although we cannot rule out the possibility that our androgen supplementation paradigms acted further up in the hypothalamo-pituitary–adrenal axis, it is unlikely that CRH or ACTH were affected because we saw no stimulatory effect of androgen on cortisol; like DHEA/S, cortisol is stimulated by ACTH but is secreted primarily from the zona fasicularis (ZF) rather than the ZR. Also, several studies suggest ACTH secretion is similar in males and females (30–33). Previous studies of post-menopausal women found that long-term administration of DHEA increased DHEA production in response to ACTH (34, 35), a finding we did not replicate presently as when DHEA was administered without testosterone we observed no night-time increase in circulating DHEAS. However, our study supplemented animals for less than 1 week, which may not have been enough time to adequately increase adrenal sensitivity to ACTH. Thus, it is likely that increased levels of both DHEA/S and T can induce the adrenal glands to produce more DHEA/S.

One endogenous disturbance of normal androgen interactions can be seen in the case of polycystic ovarian syndrome (PCOS), in which women exhibit high levels of both testosterone and DHEA/S, and these women demonstrate an increased responsiveness of DHEAS production when stimulated with ACTH (36). High levels of testosterone have in fact been implicated as a potential cause of PCOS (37, 38); however, the impact of increased testosterone on circulating DHEA/S in women has yet to be studied extensively.

One of the most common complaints in aging is a decline in cognition (39, 40), a domain that has been studied extensively with regard to estrogens, T, and DHEA/S. While some success has been seen with estrogen replacement in younger women post-ovohysterectomy (41), and with testosterone supplementation in elderly men (42–44), results from large-scale HT studies are bleak at best (45, 46). Despite promising results in rodents and benefits of the HT on other target tissues, such as maintenance of muscle mass (47), bone density (48, 49), and immune function (50, 51), DHEA/S supplementation in elderly humans (52–56) shows little to no effect on cognition. A potential drawback of all of these studies is their focus on just one component of the endocrine system. As these hormones interact, changing one steroid may result in compensation by others, rendering the effects null. Therefore, a combination of adrenal and gonadal HT may be more promising for the cognitive domain than HT with any one steroid alone.

Our results indicate that not only does the interaction between DHEAS and T occur in the adrenal gland (and not the hypothalamus, as higher ACTH would increase circulating cortisol), but it is limited to an effect on the ZR, the area that produces DHEA/S, and not the ZF, the area that synthesizes cortisol, as cortisol levels were not affected by T administration. Additionally, DHEAS, but not cortisol, decreased with age, suggesting changes in only the ZR occur with age. This is consistent with studies showing regression of the ZR in older humans (27, 57) and rhesus macaques (5). However, the ability of the adrenal gland to respond to T administration with youthful production of DHEAS suggests DHEA/S production itself is not impaired with age, but the responsiveness of the ZR to ACTH may be dampened. Thus, it may not be necessary to supplement with DHEA itself to increase circulating DHEAS levels in the elderly; merely supplementing with T with the physiologically correct time course may help to restore an overall youthful hormonal profile in elderly men.

## Author Contributions

Krystina G. Sorwell contributed to the study design, data collection, data analysis, interpretation of results, and writing and revising the manuscript. Dr. Steven G. Kohama contributed to the study design, interpretation of results, and assisted in revising the manuscript. Dr. Henryk F. Urbanski contributed to the study design, data collection, interpretation of results, and assisted in writing and revising the manuscript.

## Conflict of Interest Statement

The authors declare that the research was conducted in the absence of any commercial or financial relationships that could be construed as a potential conflict of interest.
